# Supplementary material for: 3D-printed cellular tips for tuning fork atomic force microscopy in shear mode
Source: Nat Commun. 2020 Nov 12;11:5732. doi: 10.1038/s41467-020-19536-9 (PMC7661501; doi:10.1038/s41467-020-19536-9)
Supplement: Supplementary file 1 — Supplementary Information [file 41467_2020_19536_MOESM1_ESM.pdf]

Supplementary Information

3D-printed cellular tips for tuning fork atomic force microscopy in shear mode

Liangdong Sun [1, 2], Hongcheng Gu [1, 2], Xiaojiang Liu [1, 2], Haibin Ni [1, 2], Qiwei Li [1, 2], Yi Zeng [1, 2], Ning Chang [1, 2], Di Zhang [1, 2], Hongyuan Chen [3], Zhiyong Li[1, 2], Xiangwei Zhao [1, 2, *], and Zhongze Gu [1, 2, *]

[1] State Key Laboratory of Bioelectronics, School of Biological Science and Medical Engineering, Southeast University, Nanjing 210096, China

[2] National Demonstration Center for Experimental Biomedical Engineering Education, Southeast University, Nanjing 210096, China

[3] State Key Laboratory of Analytical Chemistry for Life Science, School of Chemistry and Chemical Engineering, Nanjing University, Nanjing 210093, China

* To whom correspondence should be addressed. E-mail: xwzhao@seu.edu.cn, gu@seu.edu.cn

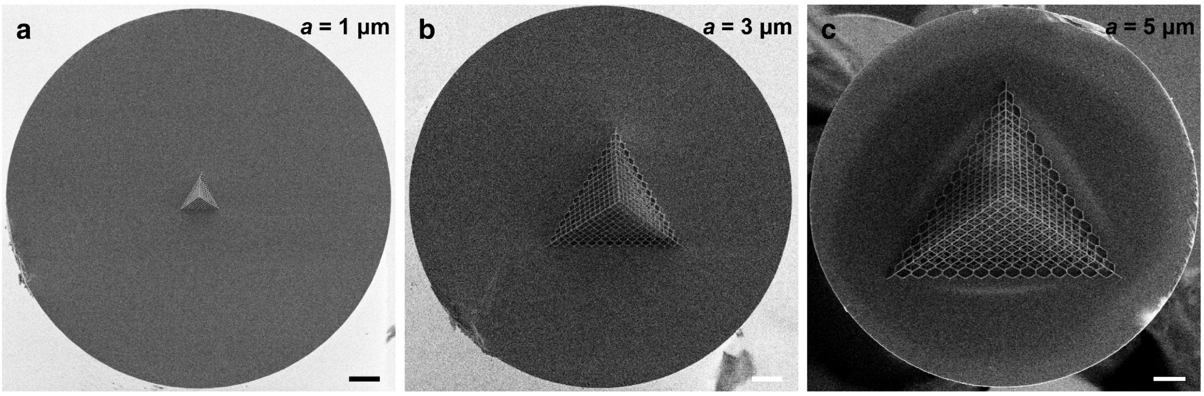

Supplementary Fig. 1 3D-printed CMA structures on fiber facets. a-c Top view SEM images of CMA structures with the unit side length of 1, 3, 5 μm, respectively. The scalar variable '*a*' at the top right corner of each figure represents unit side length. All the structures have a default 15 stacking layers of cellular units. The circular dark backgrounds underneath the structures are single-mode optical fiber facets. All scale bars are 10 μm.

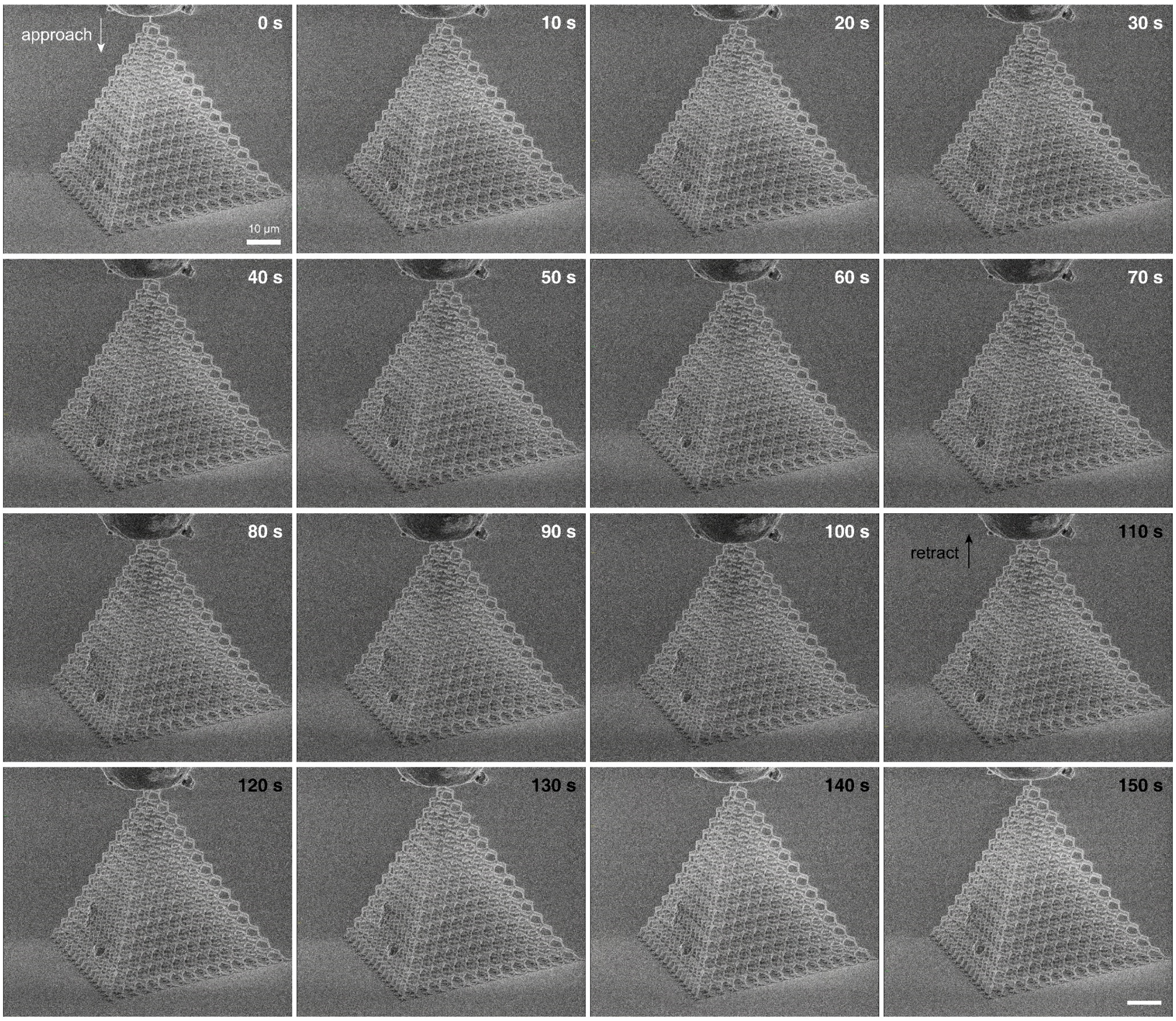

Supplementary Fig. 2 In-situ SEM characterized indentation test of CMA structure (*a* = 5 μm). The whole indentation process continues for 150 s, including approaching (0-100 s) and retracting (100-150 s) process. The time is correspondingly marked on the top right corner of each image. The scale bar is 10 μm for all images. Samples were compressed at a strain rate of 10^{-3} s^{-1}.

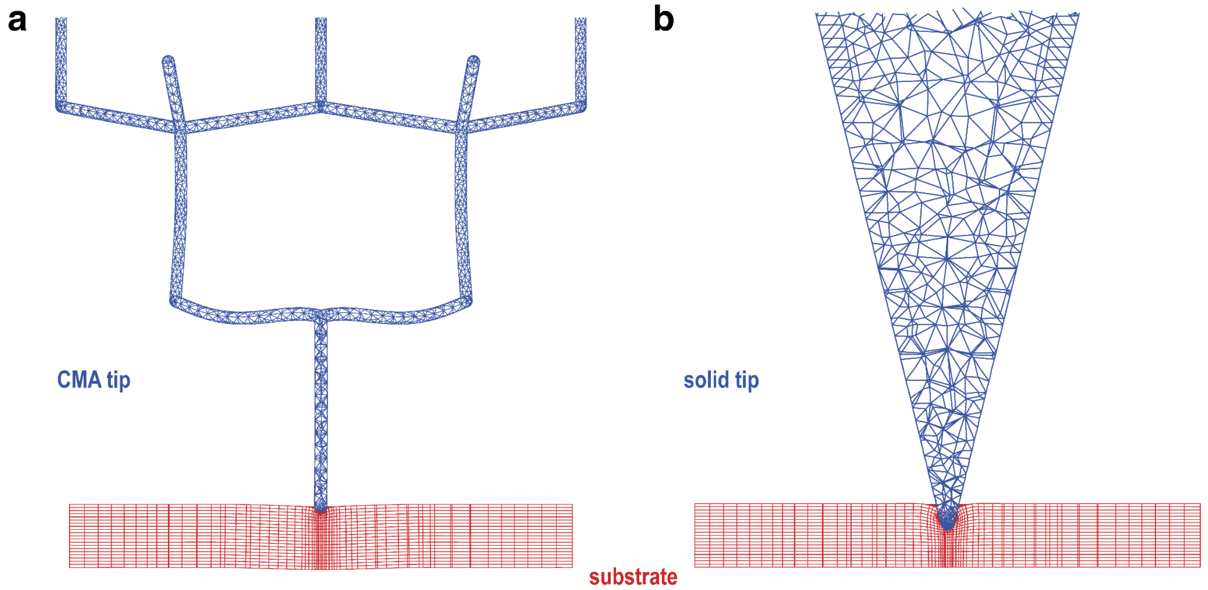

Supplementary Fig. 3 Schematics of the mechanical response of tip and substrate to dynamic impact in the scanning process. a Scanning with the CMA tip. **b** Scanning with the solid tip (cone shape). All objects are displayed in a render style of wireframes.

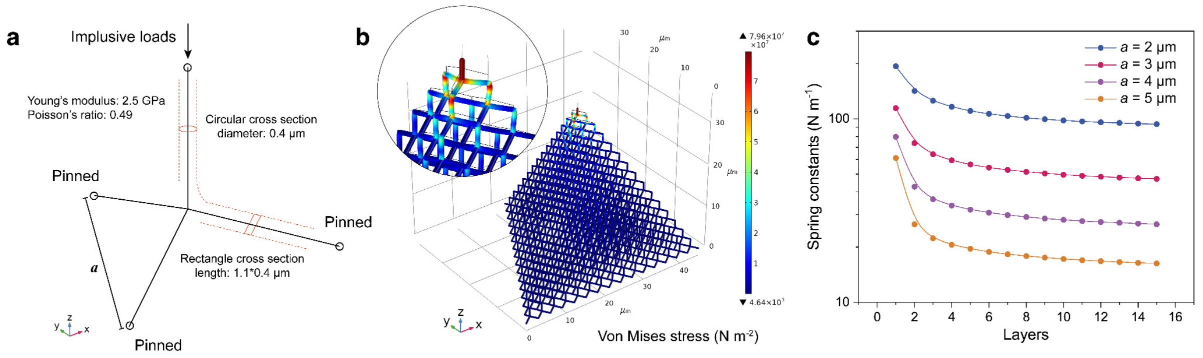

Supplementary Fig. 4 Compressive response of CMA structures based on static loading. a Model configuration and boundary condition for the simulation. The material is specified with a Young's modulus of 2.5 GPa and a Poisson's ratio of 0.49. The cross-sectional settings for struts are marked in the schematic to approach the manufactured shape. **b** Deformation and stress distribution of CMA structure (a = 3 μm), when 10 μN is applied on its apex in -z-

direction. **c** Tunable spring constants range of CMA structures as a function of stacking layer numbers. Source data of (**c**) are provided as a Source Data file.

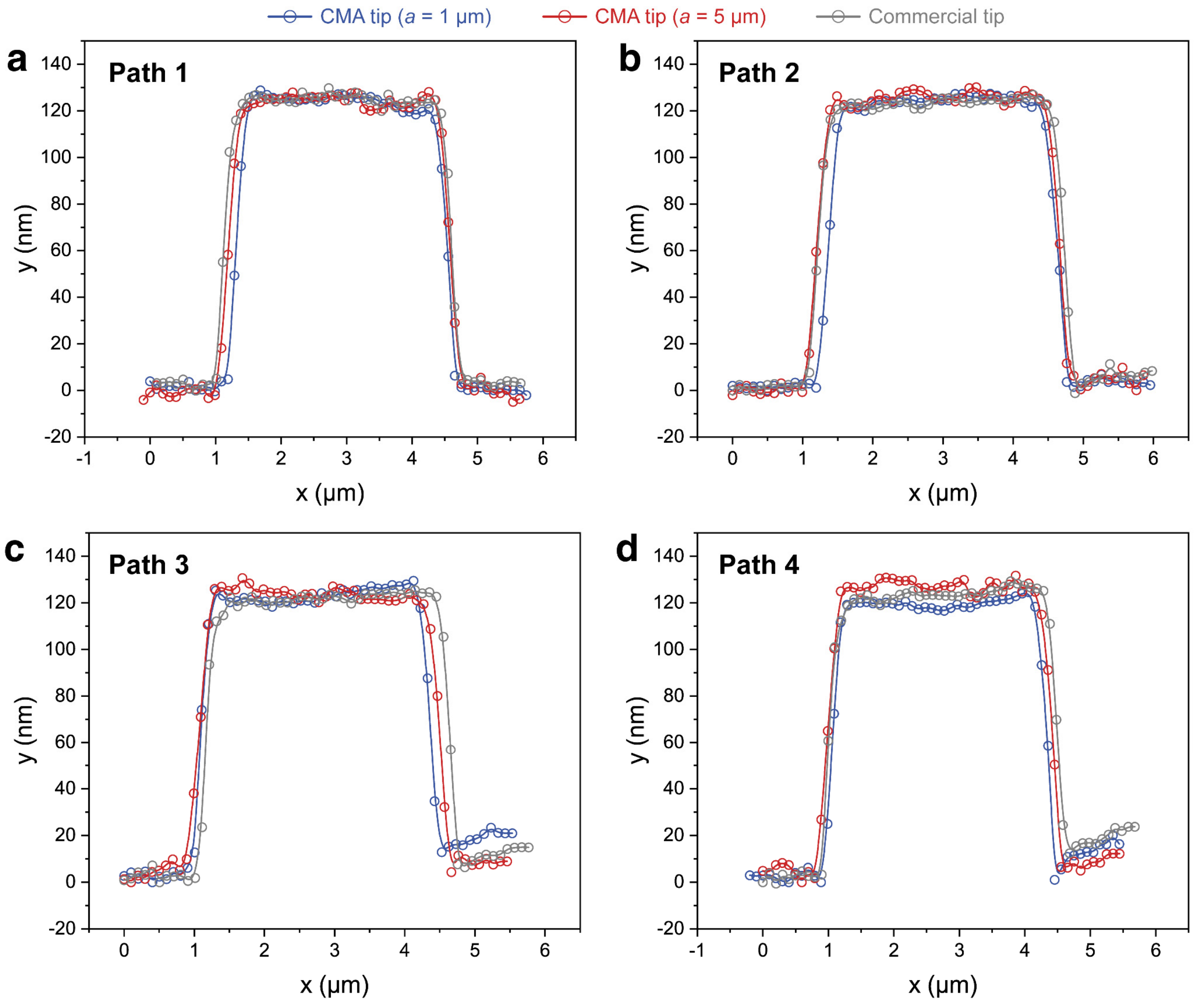

Supplementary Fig. 5 Height profiles of step pattern on silicon microgrid acquired by different tips. a-d Height profiles along path 1-4 (marked in manuscript Fig. 3a), respectively. All the plots share the same figure legend. Source data of (**a-d**) are provided as a Source Data file.

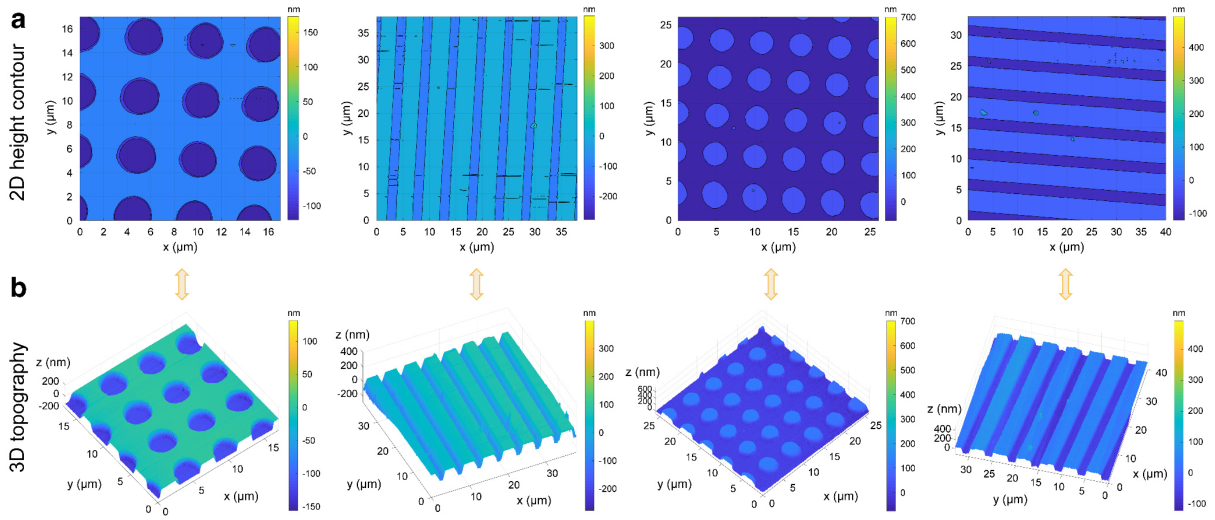

Supplementary Fig. 6 AFM images of silicon microgrid with the CMA tip (*a* = 1 μm). a 2D height contour plots. **b** Corresponding 3D topographies.

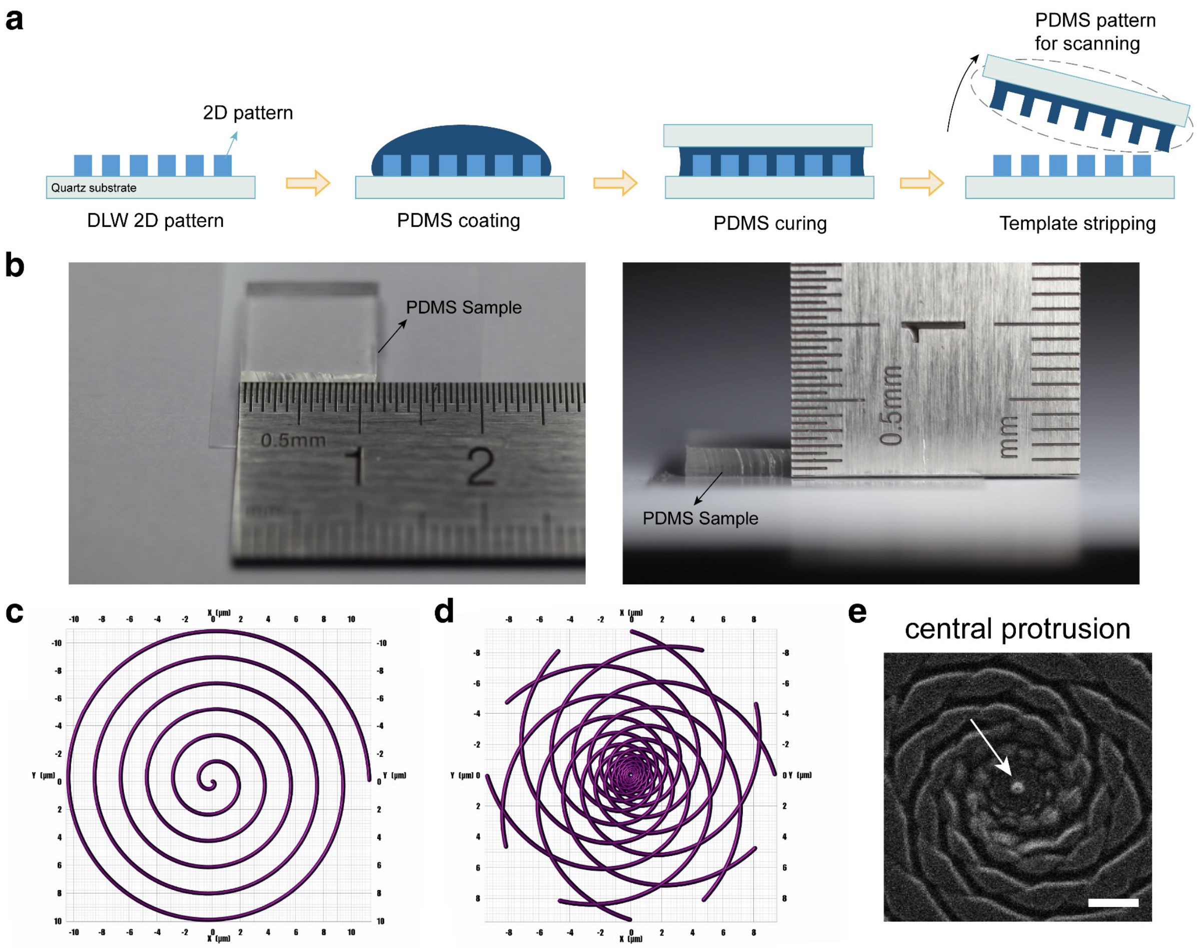

Supplementary Fig. 7 Design and fabrication of PDMS mold for scanning. a Schematics of pattern transferring processes from DLW template to PDMS mold by template stripping method, including DLW 2D pattern, PDMS coating, PDMS curing, template stripping. **b** Photographic images and size information of PDMS sample employed for scanning. **c, d** Template designs of spiral and flower patterns for DLW, respectively. **e** SEM image of fabricated PDMS flower pattern with a white arrow indicating the central protrusion. The scale bar is 2 μm.

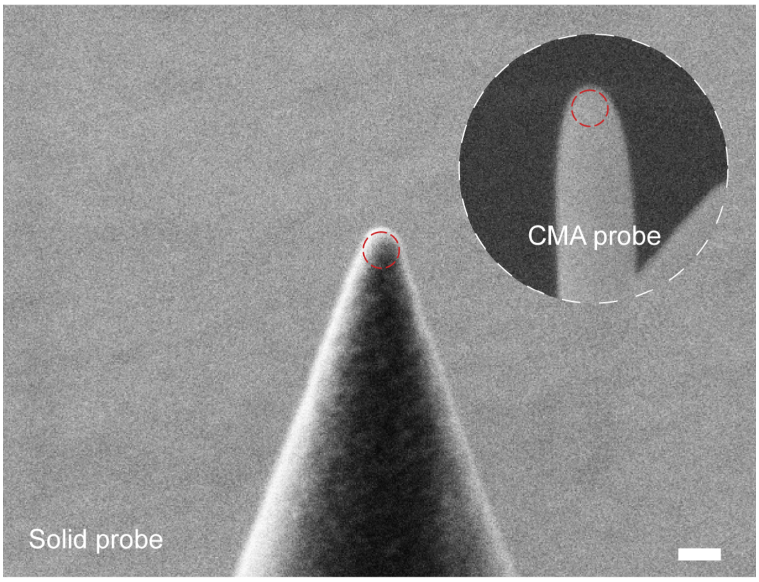

Supplementary Fig. 8 SEM images of the apexes of the solid tip and CMA tip (inset picture). The red dashed circles have an equal size. Scale bar is 200 nm for both the main image and the inset.

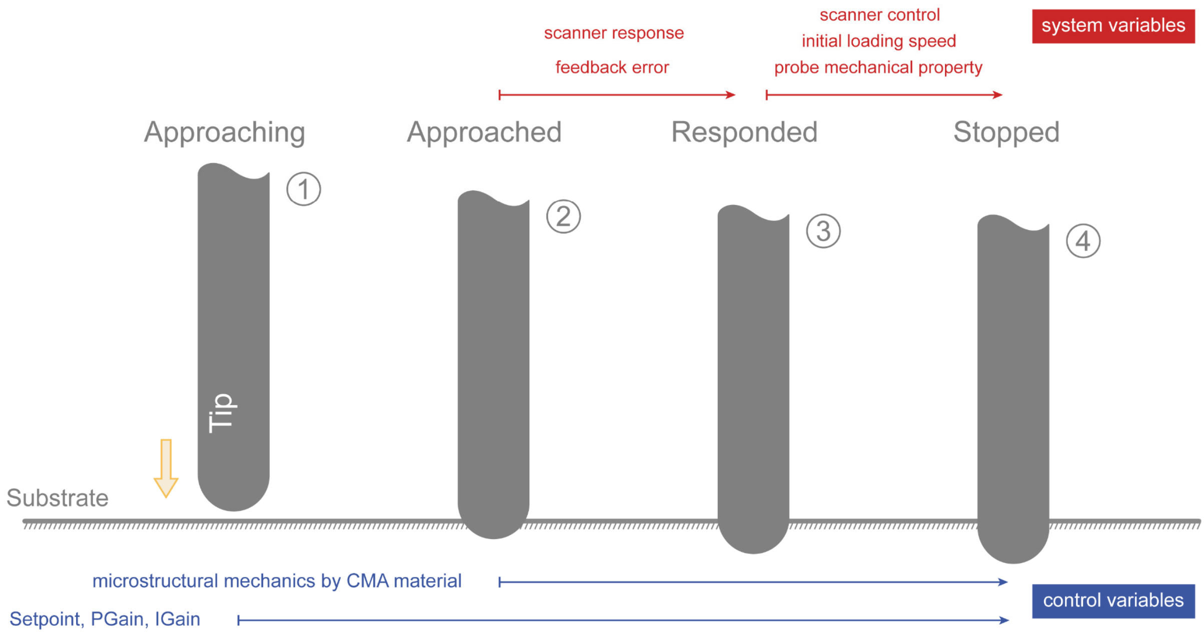

Supplementary Fig. 9 Schematic of the tip impact process. The process is composed of four steps (i.e., approaching, approached, responded and stopped). The red items indicate system variables that affect the impact process while the blue items show the corresponding adjustable variables that control the impact process. The arrowed lines indicate the actuation duration for the variables.

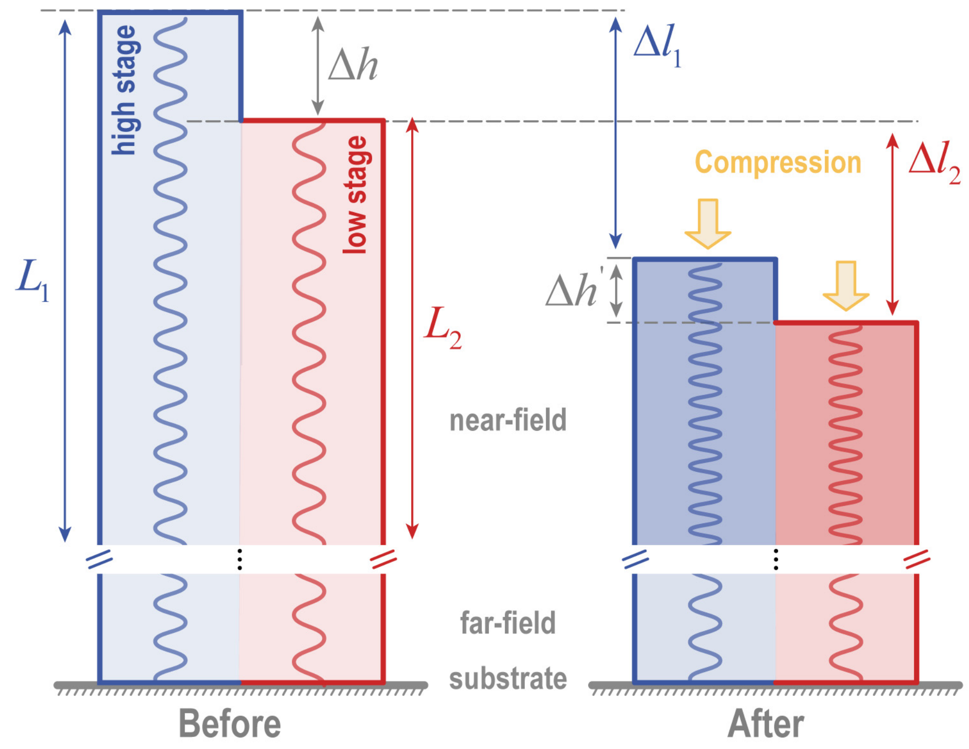

Supplementary Fig. 10 Schematic of the transient near-field response for the high (blue) and low (red) stage of a PDMS step pattern before and after the impact compression. This model assumes that material under the tip impact can be isolated from the surroundings and treated as springs (marked as wavy lines in the schematics) in the calculation[1].

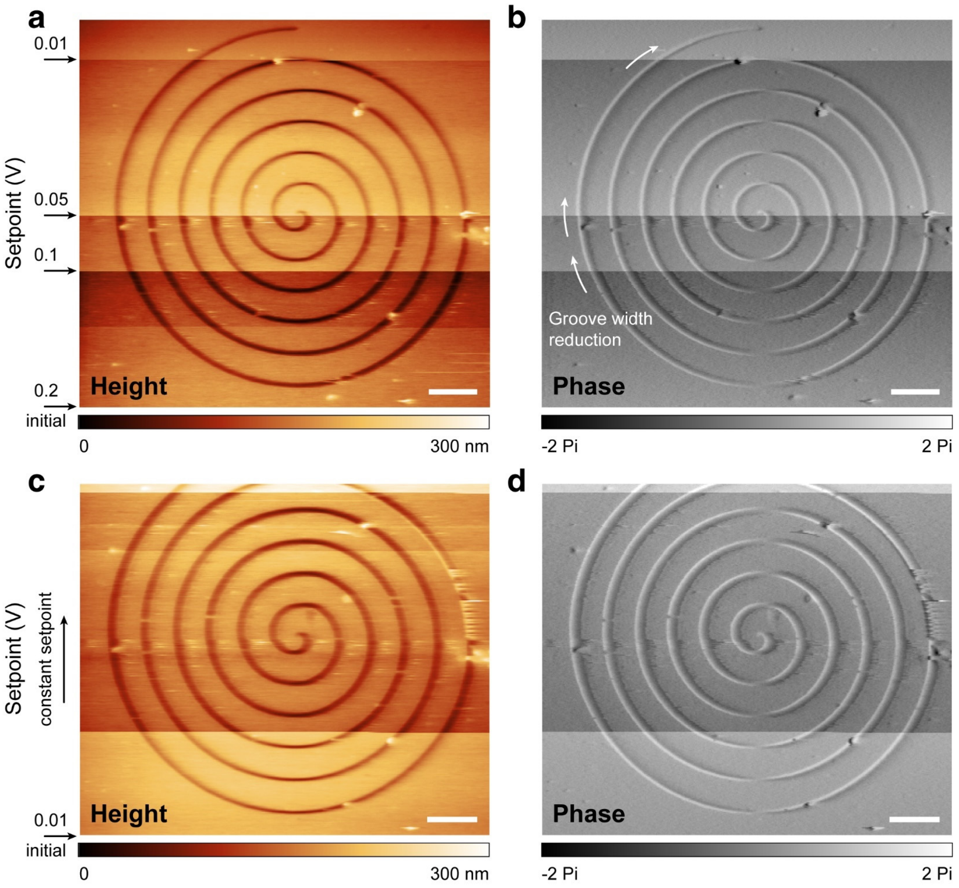

Supplementary Fig. 11 Height and phase imaging of PDMS spiral pattern with a commercial probe by adjusting setpoint values. a, **b** Obtained height and phase details by gradually decreasing setpoint from 0.2 to 0.01. **c**, **d** Height and phase images at a constant low setpoint value of 0.01. All scale bars are 3 μm.

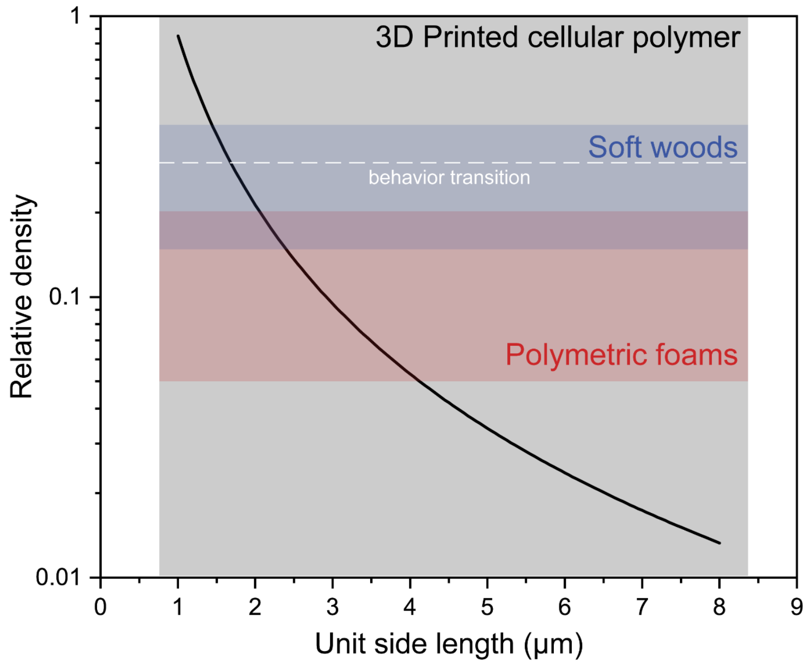

Supplementary Fig. 12 Calculated relative density of CMA structures as a function of unit side length.

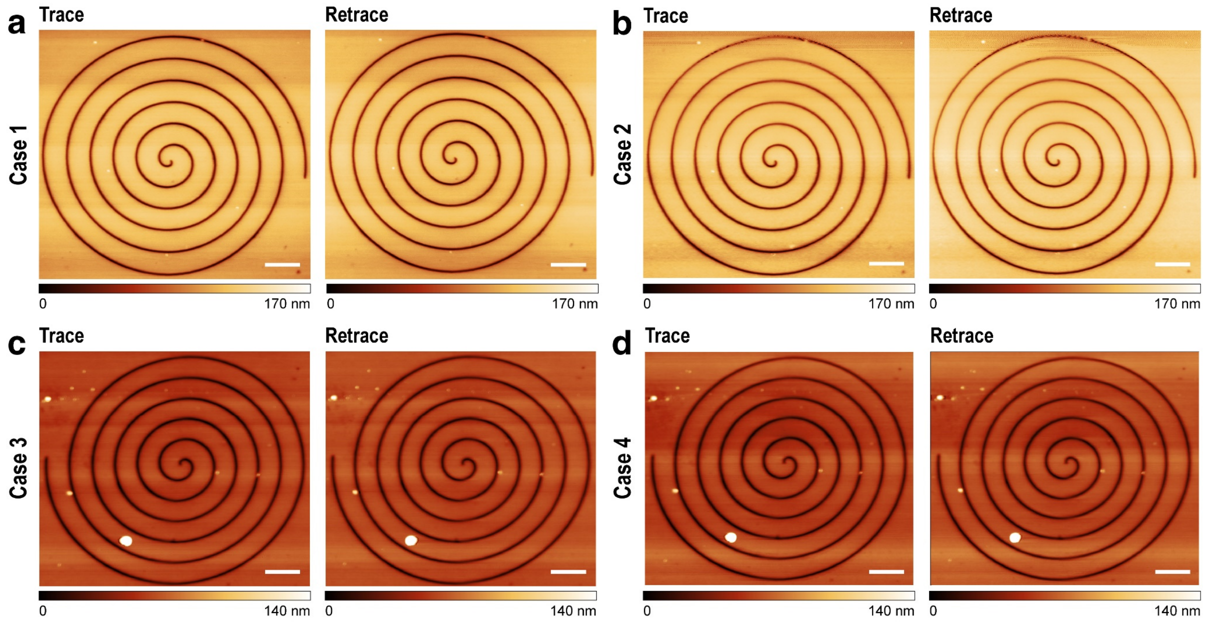

Supplementary Fig. 13 Height images of PDMS spiral patterns by CMA tips (*a* = 5 μm). a-d Height images obtained from trace and retrace processes using four different CMA tips (Case 1-4). All scale bars are 3 μm.

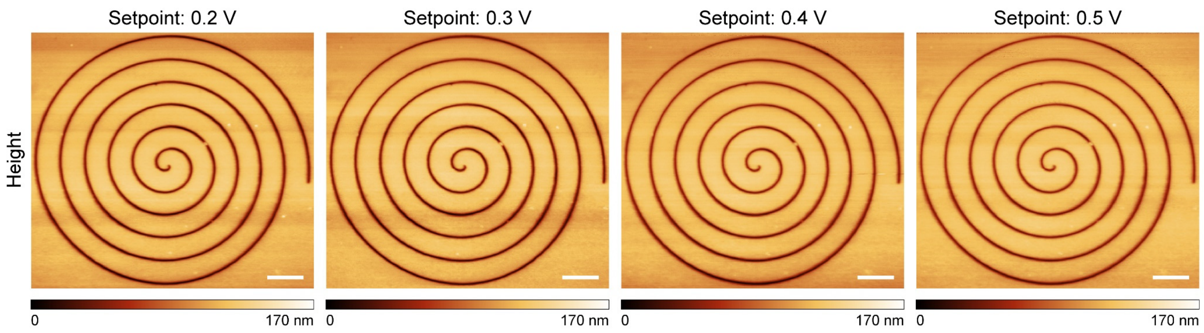

Supplementary Fig. 14 Height images of PDMS spiral patterns acquired by CMA tips (*a* = 5 μm) at increasing setpoint values. All scale bars are 3 μm.

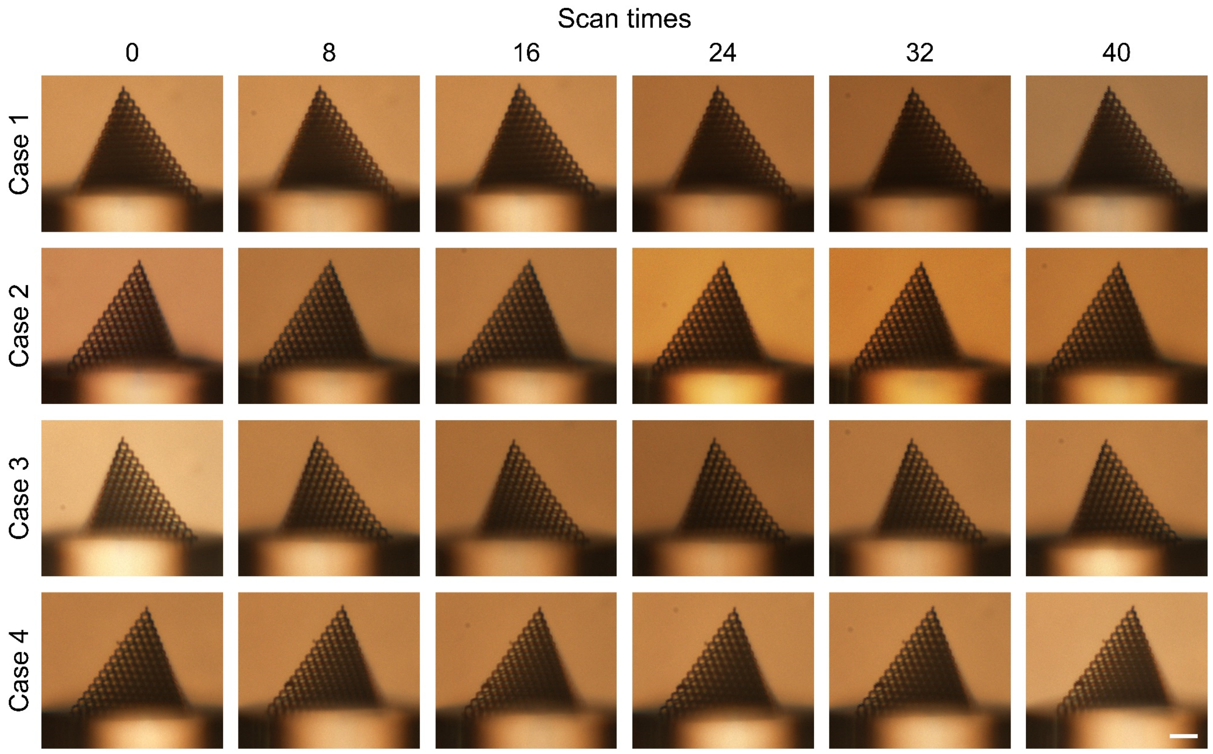

Supplementary Fig. 15 Durability test of CMA tips. Four CMA tips (Case 1-4) were employed for the wear tests by repeated scans (40 times) on PDMS mold. The scale bar is 15 μm for all images.

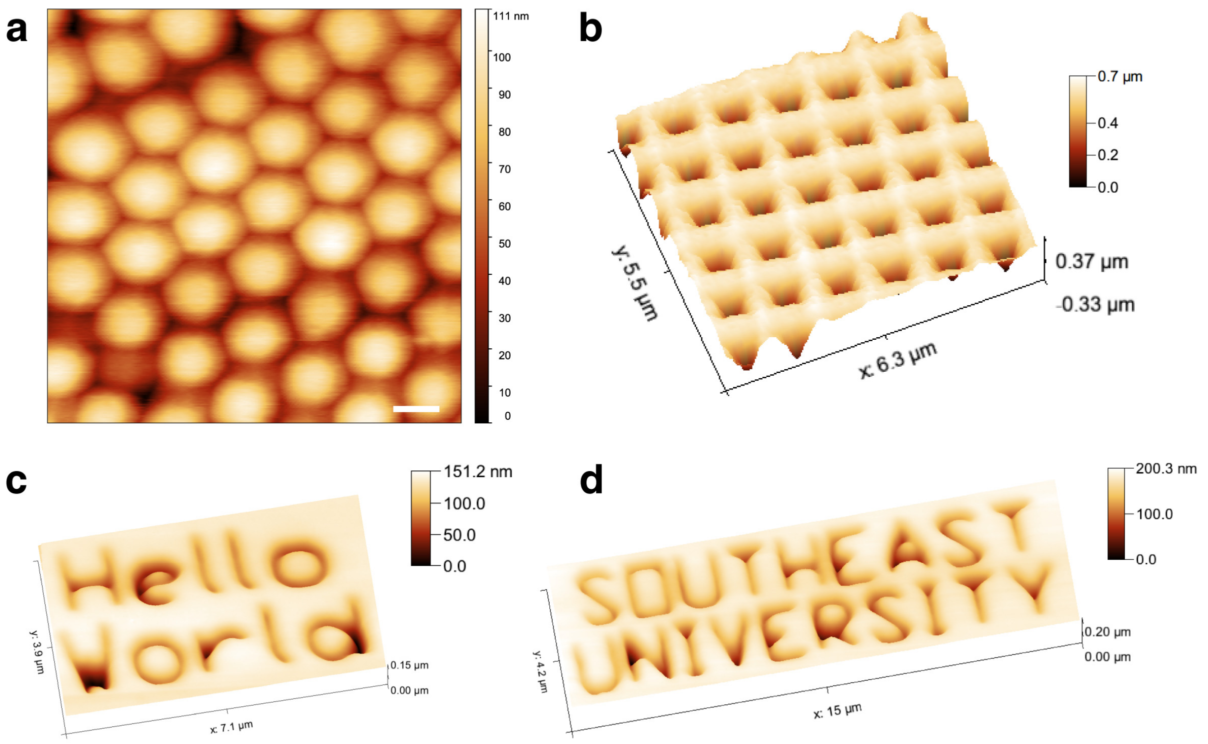

Supplementary Fig. 16 AFM imaging of soft samples using the CMA tip (*a* = 5 μm). a Polystyrene nanoparticles. The scale bar is 200 nm. **b-d** PDMS molds.

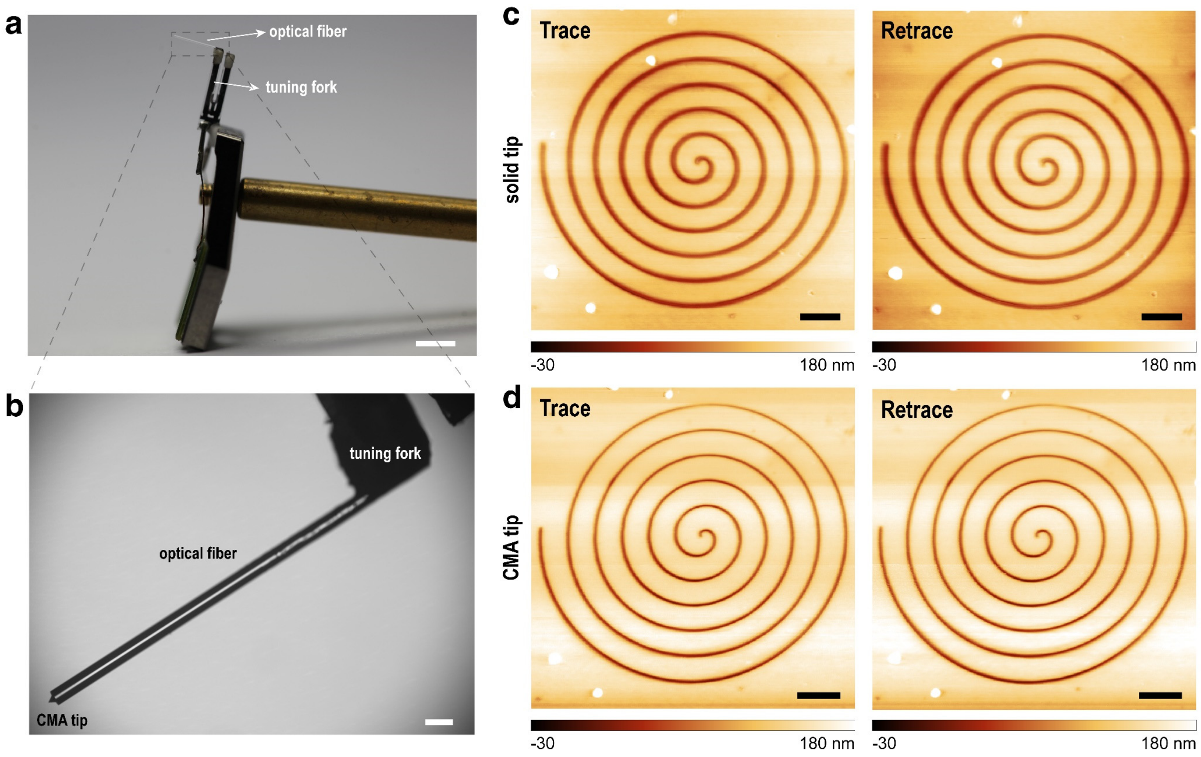

Supplementary Fig. 17 Normal-mode imaging with CMA tips. a, **b** Photographic image of assembled normal-mode probe based on cantilevered tuning fork and the optical micrograph of its components, respectively. Scale bars are 2 mm for (**a**) and 200 μm for (**b**). **c**, **d** Height images obtained from trace and retrace processes with solid tip (solid cone) and CMA tip (a = 5 μm), respectively. All scale bars are 3 μm for (**c**, **d**).

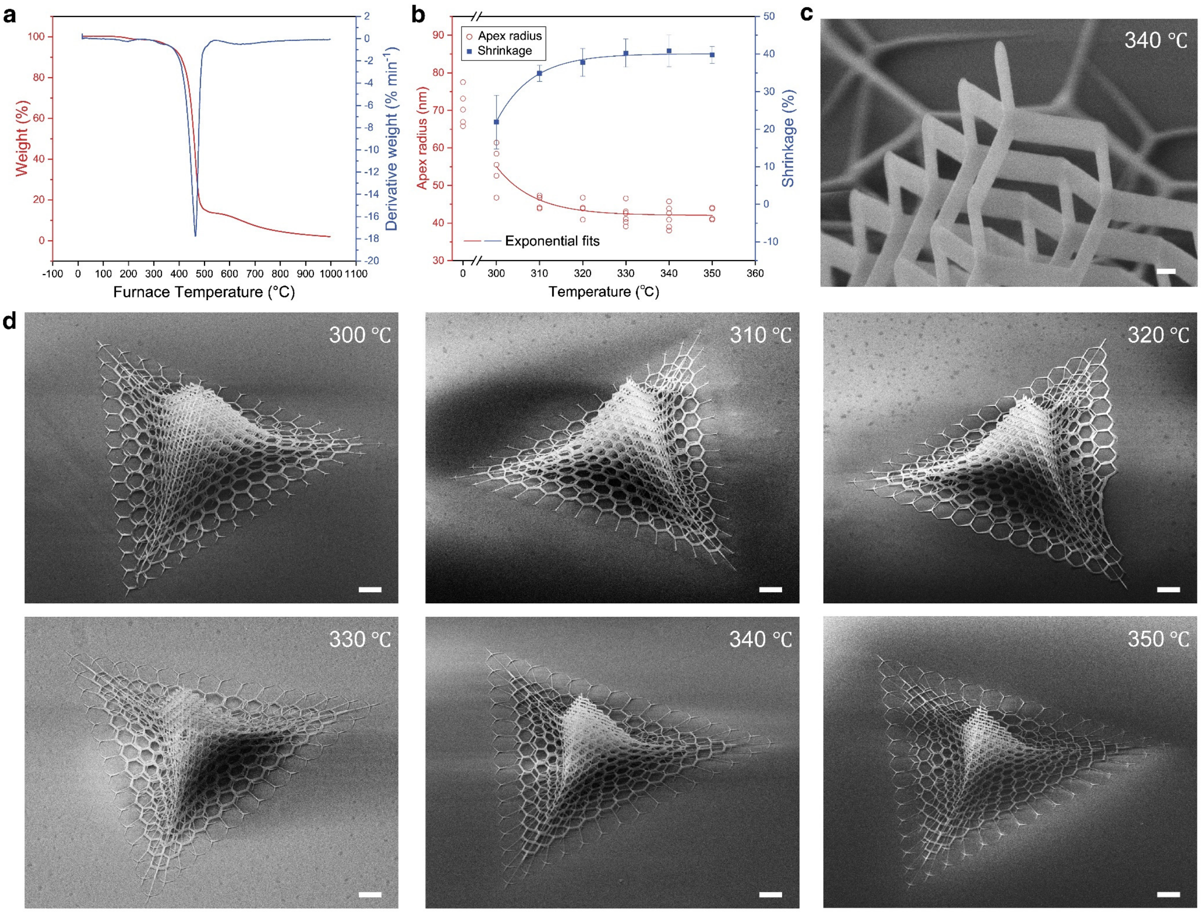

Supplementary Fig. 18 Pyrolysis of CMA structures (*a* = 5 μm). a Thermogravimetric analysis of the constituent material (cured photoresist IP-Dip polymer) of CMA structure in a nitrogen atmosphere. **b** Apex radius and calculated shrinkage as a function of max furnace temperature. **c, d** SEM images of pyrolysis products at different maximum temperatures. The maximum temperature values are marked at the top right corner of corresponding images. Scale bars are 300 nm for (**c**) and 5 μm for (**d**). The error bars in (**b**) are standard deviation. Source data of (**a**, **b**) are provided as a Source Data file.

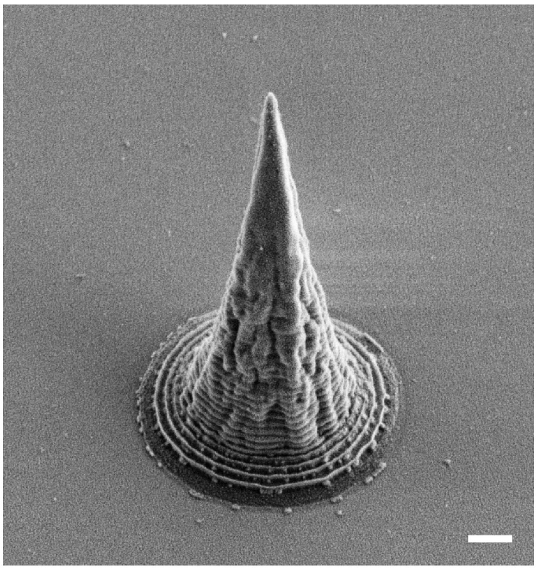

Supplementary Fig. 19 Pyrolysis of a 3D-printed solid cone. The Scale bar is 1 μm.

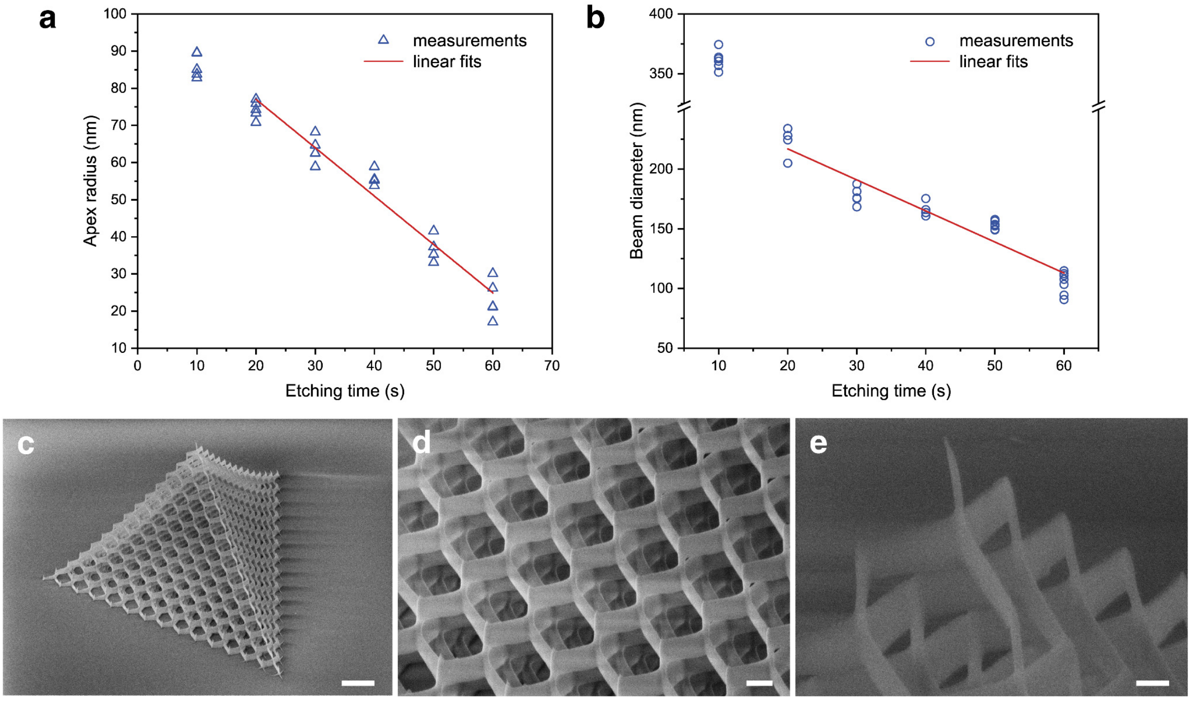

Supplementary Fig. 20 RIE (reactive ion etching) machining of the CMA tip (*a* = 5 μm). **a**, **b** Measured apex radius and vertical beam diameter as a function of etching duration, respectively. **c-e** SEM figures of CMA structure after 60 s RIE. The apex size in **e** is 17 nm.

Scale bars are 5 µm, 1 µm, 500 nm for (**c**-**e**), respectively. Source data of (**a**, **b**) are provided as a Source Data file.

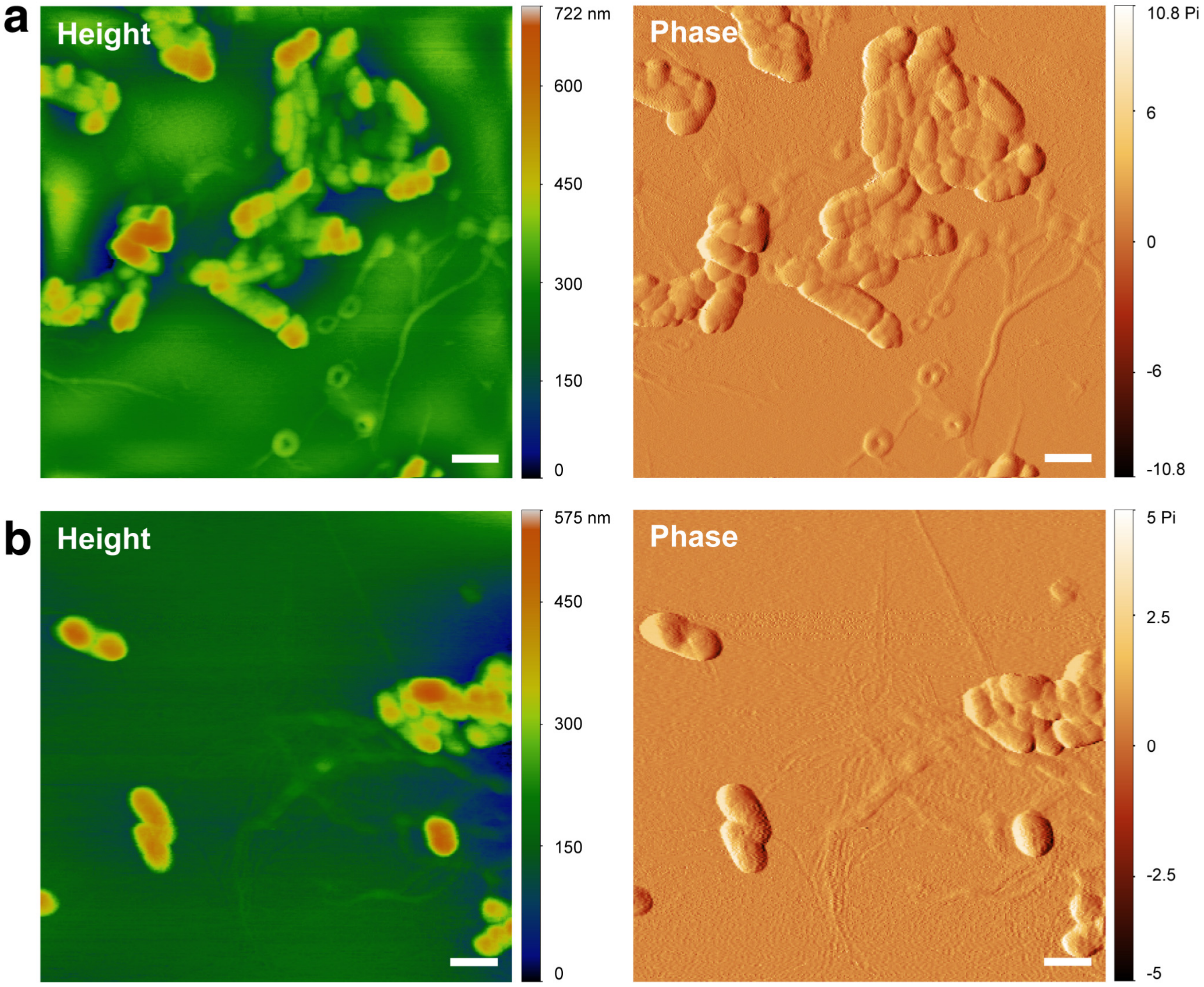

Supplementary Fig. 21 Height and phase images of Shewanella MR-1 bacteria cells using an RIE-etched CMA tip (*a* = 5 µm). All scale bars are 2 µm.

Supplementary Tables

Case	Resonance Frequency Before/After	Q Factor Before/After
Case 1	39.29/34.11 kHz	1925/854.4
Case 2	36.72/35.62 kHz	1945/256.4
Case 3	39.89/34.21 kHz	1415/366.77
Case 4	39.67/36.09 kHz	1200/225.34

Supplementary Table 1 | Measured resonance frequency and Q factor of CMA probes (*a* = 5 μm) before and after mounting.

Case	Resonance Frequency	Q Factor
Commercial probe (silicon microgrid imaging)	33.08 kHz	1795
CMA probe (a = 1 μm) (silicon microgrid imaging)	34.1 kHz	254.41
CMA probe (a = 5 μm) (silicon microgrid imaging)	33.61 kHz	323.3
Solid probe (PDMS patterns imaging)	35.64 kHz	345.3
CMA probe (a = 5 μm) (PDMS patterns imaging)	32.41 kHz	287.2
CMA probe (a = 2 μm) (PDMS patterns imaging)	34.23 kHz	342.5
Etched CMA probe (a = 5 μm) (cell imaging)	35.49 kHz	269.2
Commercial probe (cell imaging)	37.48 kHz	920

Supplementary Table 2 | Mechanical parameters of relevant probes used in the experiments.

Supplementary Note

Supplementary Note 1: Mechanical properties of CMA probes.

The CMA body is built on top of an optical fiber end face, which is subsequently bond to the tuning fork for AFM imaging. Additional mass will undoubtedly degrade the mechanical performance of the tuning fork. To investigate the aspect, we randomly chose four tuning forks and conducted the assembly process. Here, the fibers with CMA structures on one end were carefully cut into ~3-5 mm pieces, placed on one arm of the tuning fork and immobilized using UV-adhesive. The resonance frequency and quality factor measured using the AFM system are recorded in Supplementary Table 1. The resonance frequency of the four cases decreases by 1.1-5.6 kHz, 3%-14% drops compared to its original resonance frequency before mounting. However, the variations of Q factors are much more significant, with 55.6%-86.8% decreases. In practical experiments, low Q factors have not yet shown a significant degradation of image quality. Conversely, the enhanced damping properties could be utilized for high-speed imaging since the detection speed is proportional to the ratio of resonance frequency to quality factor[2-4].

Supplementary Note 2: Static compressive response of CMA structure.

Finite-element simulations based on static loading was performed to predict the stiffness of the CMA structures. In order to approach the fabricated shapes via DLW, SEM determined beam sizes were applied to specify axial cross-sections for four beams in each cell, as marked in Supplementary Fig. 4a. The feet of bottom cells are attached to the ground with constraints in all dimensions. The structure deforms in the presence of a force (1-10 μN) uniaxially applied

to the apex in -*z*-direction compressing the structure. The distribution of stress and deformation is illustrated in Supplementary Fig. 4b. The stress rapidly attenuates when passing through the lattice layers, suggesting that the underlying cells are underused, and solely adjusting layer numbers is not an effective method to tune its mechanical property. As shown in Supplementary Fig. 4c, spring constants remain almost unchanged when the stacking layers exceed a certain amount (e.g., 10 layers). Therefore, the stiffness tuning of the CMA structure generally relies on the control of the lattice size.

Supplementary Note 3: Apex comparison between CMA tip and solid tip

To ensure the imaging difference by the CMA tip and the solid tip is dominated rather by the microarchitectural construction than the apex, both apexes are observed on SEM. As seen in Supplementary Fig. 8, the apex sizes indicated by red circles are dimensionally identical, albeit in different structural constructions. Fabricated via the same DLW technique, both apexes are composed of one single voxel, the size of which is only governed by laser output and scan speed, finally leading to a similar size by strictly controlling these parameters constant in the whole manufacturing process. The apex radius is measured to be 86±2.9 nm.

Supplementary Note 4: Analysis of the impact process

To better clarify what role CMA material may have in the scanning, the impact process is divided into four steps, as illustrated in Supplementary Fig. 9. After the approaching step is initiated, in the second step, the tip will subsequently contact the surface determined by preset setpoint value. However, the scanner would not stop immediately by the time it receives the

in-contact signal. Specifically, the integrator gain parameter sets the time for the scanner to respond for any change of the error signal of setpoint. During this short period between step 2 and step 3, the scanner would keep moving downward until the feedback is responded, and the tip is then decelerated until it finally stopped in step 4. Here, in addition to the necessary response time required by the hardware settings, feedback errors, including signal hysteresis and dysfunction, would significantly extend this duration. The final deceleration is mainly controlled by the scanner parameter settings, the loading speed of the tip, and its mechanical property. Corresponding to all the listed system variables that affect the impact process, the control feedback parameters (i.e., setpoint, PGain and IGain) and the application of energy-absorbing CMA material are two main adjustable variables to control the impact process. The setpoint determines the amount of error signal changes of a tuning fork (phase or amplitude) in the approaching process, which returns a specific contact state, directly correlating to the force strength applied on the surface and indirectly affecting approaching speed. Increased setpoint and gain parameters would increase the approaching speed of the tip and thus enhance the contact interaction. Hence, feedback parameter control is functioning in all four steps, which are strictly kept the same in the comparison experiments. Even though the contact force in step 1-2 is independent of tip selections by designating the equal value of setpoint, the positive buffer effect provided by the CMA tip still works effectively in the rest steps. The cellular lattices serving as sacrificial layers to absorb the kinetic energy of the impulse during the response and deceleration period rapidly decelerate the tip, which finally contributes to the mitigation of impact as well as surface deformation.

Supplementary Note 5: Estimation of interfacial stress level in the dynamic impact of tips

The impact dynamics[5] generally features a temporal overpressure[6] closely localized in the interaction interface at the beginning of the impact, which decays with interaction period and propagating distance[7-10]. Within the near-field region[6], the overpressured stress can be approximately treated with a uniform distribution, leading to a significant difference of indentation displacement in the compression of an uneven surface. Therefore, as an average approximation in this interaction duration as well as the effective dimension of local compression, the transient stress in the deceleration period of tips can be determined from the distortions of imaged patterns. Based on the difference of imaging outcomes by the CMA tip and the solid tip as revealed in Fig. 4f the stress level could be quantitively compared by introducing the measured depth of the nanochannel into a comressive spring model.

Considering the same stress is applied to compress both higher and lower stages of a typical step morphology, the material in the near-field of the impact can be isolated from the surroundings and treated as springs with different initial lengths in calculation[1], as schematically given by Supplementary Fig. 10. Therefore, the measured height difference between the stages could be represented by

$$\Delta h' = (L_1 - \Delta l_1) - (L_2 - \Delta l_2) \qquad (1)$$

where L_1 and L_2 represent the original height of the upper and lower stages, respectively, and Δl_1 and Δl_2 denote corresponding displacements. Since sample modulus (M) is derived from the ratio of stress σ and strain ε, the stress can be expressed as

$$\sigma = M\varepsilon = M\Delta l_1/L_1 = M\Delta l_2/L_2 \qquad (2)$$

and the displacement could be determined as

$$\Delta l_1 = \sigma L_1 / M \qquad (3)$$

$$\Delta l_2 = \sigma L_2 / M \qquad (4)$$

Therefore, the measured height difference could be expressed as

$$\Delta h^{'} = (L_1 - \Delta l_1) - (L_2 - \Delta l_2) = (1 - \sigma/M)(L_1 - L_2) = (1 - \sigma/M)\,\Delta h \qquad (5)$$

$$\Delta h = L_1 - L_2 \qquad (6)$$

where $\Delta h^{'}$ denotes the measured value by tips in the scanning, while Δh denotes the real value. Therefore, the stress level could be deduced from formula (5) as follows:

$$\sigma = M(1 - \Delta h^{'}/\Delta h) \qquad (7)$$

Therefore, the stress ratio between tips can be further determined from the measured height:

$$\sigma_{\mathrm{CMA}}/\sigma_{\mathrm{solid}} = (\Delta h - \Delta h^{'}_{\mathrm{CMA}}) / (\Delta h - \Delta h^{'}_{\mathrm{solid}}) \qquad (8)$$

where σ_{CMA}, σ_{solid} represent the stress from CMA tip and solid tip, and $\Delta h^{'}_{\mathrm{CMA}}$, $\Delta h^{'}_{\mathrm{solid}}$ correspond to the measured height by CMA tip and solid tip, respectively. Calibrated by a new scan on BioScope Resolve™ of Bruker (tip: NP-S10A, k: 0.58 N m^{-1}, setpoint peak force: 100 pN), the real height of the spiral channel is measured to be 85.9±3.1 nm. Substituting for the parameters with $M = 2.6$ MPa, $\Delta h^{'}_{\mathrm{CMA}} = 83.6 \pm 3.3$ nm and $\Delta h^{'}_{\mathrm{solid}} = 67.9 \pm 8.2$ nm and considering the random interfacial stiffness distribution suggested by roughness measurement, the average local stress exerted by the CMA tip (a = 5 μm) is approximately 0.07 MPa, which is about 9.8%-18.3% compared to stress produced by the solid tip. According to the calculated stress levels, the local strain in the interfacial region induced by CMA tips is about 0.027 while the value is raised to 0.148-0.276 for the solid tip.

Please note the calculation supported by the compressive spring model is based on an ideal situation that ignores the possible slight change of interfacial stiffness distribution and impact

propagation in the nanoscale caused by dynamic impact. Furthermore, the approximated stress and corresponding strain are transient quantities localized in the near-field (Supplementary Fig. 10) underneath the impact within the milliseconds' duration of deceleration phase, which do not retain an absolute correspondence with the global mechanical response as in the case of static or quasi-static loading. Certainly, the impact-induced stress waves kept propagating to the far-field as the tip was decelerated and stopped, causing a further deformation. However, the overpressured stress wave is not likely to constantly continue spreading to the global causing a macroscopic deformation, since, on one hand, the overpressured stress keeps fast attenuating with the propagating distance and interfacial stretching contributing to far less displacement than in the near-field, on the other hand, no energetic stress is further produced any longer to continuously support the entire compression in the global scale as the tip finally stopped. Based on the calculated stress level, numerical analysis, approaching speed as well as impact duration, the maximum local indentation depth for solid tips corresponding to micron-scale compressible dimension will be hundreds of nanometers approximately depending on the actual approaching speed, which coincides with all the experimental results. The CMA tip has a much smaller indentation depth due to its cushioning property, yielding more accurate and reliable images without excessive mechanical interactions.

Additionally, given a macroscopic sample thickness of about 1.75 mm (Supplementary Fig. 7b)[1], the PDMS sample is better regarded as a bulk polymer solid. The influence on imaging quality from sample thickness can be ignored, since, on one hand, the impact-induced local displacement of the sample based on the above-mentioned impact dynamics is roughly four orders of magnitude less than the global scale, on the other hand, all the imaging

experiments of the PDMS patterns were strictly performed on the same PDMS sample and quartz slide underneath, which further excludes the existence of sample thickness as a potential variable affecting the imaging quality.

Supplementary Note 6: Supplemented imaging tests on PDMS patterns by a commercial probe

The commercial probe (used in the imaging tests of silicon microgrid) was applied to scan the PDMS spiral pattern. The setpoint value was tuned to verify if the parameter tuning could replace the CMA probe and improve imaging quality. In Supplementary Fig. 11a,b, the gradually decreased setpoint (from 0.2 to 0.01), which is intended to reduce the interaction force exerted by the tip, correspondingly leads to a gradual reduction of groove width shown in the phase plot, indicating the successful mitigation of local stresses. Therefore, a new test was initiated with a constant setpoint value of 0.01, which is typically the noise level of error signals. However, pushing the sensitivity to such an extreme limit is at a cost of scanning stability and accuracy. As given by Supplementary Fig. 11c,d, the groove size retains well at the beginning of the scanning course but soon increases with significant image degradation. The imaging process experiences undershoots and overshoots especially when crossing steep obstacles and finally fails due to feedback loss. In contrast, inspired by the similar height images at increasing setpoint values presented in Supplementary Fig. 14, the imaging by CMA tips seems to be more independent of fluctuation in stress level or feedback parameters. In the whole tests using commercial probes, the same image quality realizable via the CMA probes was never obtained at any tuning combinations of feedback parameters, which demonstrates

the irreplaceability of CMA probes in imaging optimization.

Supplementary Note 7: Durability tests of CMA tips

To investigated the wear characteristics, CMA tips (a = 5 μm) were employed for repetitive scans of total 40 times on PDMS samples. Each scan has two cycles (i.e., trace and retrace), and each scans 200×200 points with each point lasting 5 ms, consuming 12 min for each scan. The scan parameters are purposely increased to enhance the interaction (setpoint:0.3, PGain:0.8, IGain:2.4). The scanning images are exhibited in Supplementary Fig. 13. Distinct nano features and consistent measurements between trace and retrace processes clarify the repeatability of AFM imaging using CMA tips. The tips were observed under an inverted microscope with a sequence of images taken at increasing scan times, as shown in Supplementary Fig. 15. Most tips were well-preserved after hours of continuous work. However, in case 4, a slight tilt of the apex was observed, likely arising from the shear-force mode applied in the imaging, in which case, the tuning fork vibrates in a parallel direction to the sample surface. It could be optimized by enhancing the polymerization of constituent material or changing the working mode to normal tapping after replacing the tuning fork with a typical horizontal cantilever. However, the wear performance based on current set-up can sufficiently meet the need for common use. Typically, the coarse scans used to locate the designated area will not exceed 10 times, while there remain 20-30 times for stable imaging before the tips slowly wear out. The average lifetime of the tips will last at least over a week according to our experimental experience.

Supplementary Note 8: Normal-mode imaging with CMA tips

Even though normal-mode imaging shares the same normal contact with shear-mode imaging and theoretically does not affect the energy-absorbing performance of CMA tips in practical imaging. The normal-mode imaging based on intermittent normal contact feedback[11] was still investigated in order to further experimentally corroborate the universal benefit from the application of CMA tips, as shown in Supplementary Fig. 17. The optical fiber with the microstructural architecture at one facet was carefully cut to a length of about 2 mm, which was subsequently bonded to the cantilevered tuning fork with UV-cured adhesive prior to imaging (Supplementary Fig. 17a,b). The solid tip was employed for an imaging comparison with the CMA tip. The setpoint was controlled within a moderate range of 0.25-0.3. According to the imaging results obtained from trace and retrace processes, the control group with solid tips still clearly displays a broadened groove width in contrast to the images acquired from the CMA tip (a = 5 μm), which is similar to the imaging result based on shear mode. It suggests the effective imaging improvement derived from stress mitigation in frequent contact is still realizable with CMA tips in normal-mode, which is actually independent of specific feedback mode and can be fully utilized in any intermittent contact imaging.

Supplementary Note 9: Restrictions in the application of CMA tips

The restrictions in the applications of CMA tips mainly come from the contact mode and the resolutions.

As to the contact mode, the imaging improvement using CMA tips relies on the microstructural energy-absorbing performance, which requires both deformation and recovery

of struts in the scanning process. Therefore, it is technically suitable for this intermittent-contact based imaging that enables repetitive approach-retract processes for contact feedback at extremely high frequency. However, for typical direct-contact imaging, the tips are kept pressing against the interface without recovery during the scanning process. Unlike the situation of intermittent contact, it theoretically has no dynamic approaching events in the scanning and therefore the impulsion applied to the sample interface is not significantly related to the microstructural construction of tips and the structure-derived energy-absorbing performance, in which case the implementation of CMA tip is no longer effectively applicable.

As to resolution, it is mostly affected by the apex size of the tip. The measured apexes fabricated by DLW have an average radius of 70 nm with a minimum value of 47 nm, leading to an absolute resolution of approximately the same level, while the radius of commercial tips could reach ~5-10 nm. This is a long-existing and inevitable defect inherently derived from the photolithography process of direct laser writing in tip fabrication, which, on one hand, is expected to be improved as this technology is developing, on the other hand, can be improved with post-manufacturing such as pyrolysis or reactive ion etching as proposed in Supplementary Figs 18 and 20. The tip radius can be shrunk down to 17 nm after RIE. However, the resolution mostly governed by the tip size is not directly related to the design or implementation of microstructural tips where the contribution of our study have rooted. This work is mainly focused on the demonstration of the microstructural effect of tips in precisely rebuilding a ‘tip-free’ interfacial morphology and maintaining the original resolution without further degradation instead of greatly increasing it. Therefore, the absolute resolution limit from CMA tips is not primarily concerned in this study.

Supplementary Note 10: Pyrolysis of CMA structures

In addition to tailoring the mechanics of CMA structures by structural variation through the DLW or RIE process, the transformation of constituent material has also been investigated using pyrolysis[12, 13]. The pyrolysis of polymer resins will increase the compressive strength of the structure since the material is gradually turned into carbon. In addition, the structure size will significantly shrink due to the decomposition of molecules, which partly releases the resolution restriction originating from three-dimensional lithography[14].

According to the thermogravimetric characteristics of bulk UV-cured polymer given in Supplementary Fig. 18a, the pyrolysis includes three steps of temperature increase: (i) heating up to 250°C at a ramp rate of 3 °C min^{-1} and keeping for 1 hour, (ii) increasing the temperature to 300°C at a ramp rate of 2 °C min^{-1} and keeping for 1 hour, (iii) increasing the temperature to the maximum settings at a ramp rate of 2 °C min^{-1} and maintaining for 12 hours. As the temperature was increased to 340-350 °C, the measured apex radius was reduced to an average of 42 nm, with almost 40% shrinkage (Supplementary Fig. 18b). As revealed in Supplementary Fig. 18c,d, even though the structures are pulled down to the ground as max temperature increases, the upper part keeps its original shape perfectly. The unique construction of the cellular solid decreases its contact area with the substrate. It increases the motion freedom of internal lattices, which keeps the upper part from the severe distortions during structure transformation. As a comparison, in Supplementary Fig. 19, the product of a pyrolyzed solid entity is not satisfactory. Since the base is fixed to the ground, the whole structure will endure tense interior stresses as well as surface tension forces, which tears it to the ground, twists its

3D construction, corrugates the surface, detaches the voxel layers and blunts the apex[15]. In terms of pyrolysis, cellular solids are ideal subjects[16]. With reduced tip size, the imaging resolution of CMA tips should be accordingly increased.

Supplementary References

1. Hay, J. & Crawford, B. Measuring substrate-independent modulus of thin films. *J. Mater. Res.* **26**, 727-738 (2011).
2. Adams, J. D., Erickson, B. W., Grossenbacher, J., Brugger, J., Nievergelt, A. & Fantner, G. E. Harnessing the damping properties of materials for high-speed atomic force microscopy. *Nat. Nanotechnol.* **11**, 147-151 (2016).
3. Lee, J. S. *et al.* Multifunctional hydrogel nano-probes for atomic force microscopy. *Nat. Commun.* **7**, 11566 (2016).
4. Dietrich, P. I. *et al.* 3D-Printed Scanning-Probe Microscopes with Integrated Optical Actuation and Read-Out. *Small*, 1904695 (2019).
5. Yu, T. X. & Qiu, X. Introduction to Impact Dynamics (Tsinghua University Press, 2018).
6. Dharmasena, K. P., Wadley, H. N. G., Xue, Z. & Hutchinson, J. W. Mechanical response of metallic honeycomb sandwich panel structures to high-intensity dynamic loading. *Int. J. Impact Eng.* **35**, 1063-1074 (2008).
7. Wang, S., Ding, Y., Wang, C., Zheng, Z. & Yu, J. Dynamic material parameters of closed-cell foams under high-velocity impact. *Int. J. Impact Eng.* **99**, 111-121 (2017).
8. Visser, W., Sun, Y., Gregory, O., Plume, G., Rousseau, C. E. & Ghonem, H. Deformation characteristics of low carbon steel subjected to dynamic impact loading. *Mater. Sci. Eng., A* **528**, 7857-7866 (2011).
9. Fan, J. T., Weerheijm, J. & Sluys, L. J. Dynamic compressive mechanical response of a soft polymer material. *Mater. Des.* **79**, 73-85 (2015).
10. Liao, S., Zheng, Z. & Yu, J. Dynamic crushing of 2D cellular structures: Local strain field and shock wave velocity. *Int. J. Impact Eng.* **57**, 7-16 (2013).
11. Makky, A., Berthelot, T., Feraudet-Tarisse, C., Volland, H., Viel, P. & Polesel-Maris, J. Substructures high resolution imaging of individual IgG and IgM antibodies with piezoelectric tuning fork atomic force microscopy. *Sens. Actuator B-Chem.* **162**, 269-277 (2012).
12. Gao, H. L. *et al.* Super-elastic and fatigue resistant carbon material with lamellar multi-arch microstructure. *Nat. Commun.* **7**, 12920 (2016).
13. Wilson, N. R. & Macpherson, J. V. Carbon nanotube tips for atomic force microscopy. *Nat. Nanotechnol.* **4**, 483-491 (2009).
14. Bauer, J., Schroer, A., Schwaiger, R. & Kraft, O. Approaching theoretical strength in glassy carbon nanolattices. *Nat. Mater.* **15**, 438-443 (2016).
15. Zakhurdaeva, A., Dietrich, P.-I., Hölscher, H., Koos, C., Korvink, J. & Sharma, S. Custom-Designed Glassy Carbon Tips for Atomic Force Microscopy. *Micromachines* **8**, 285 (2017).
16. Burckel, D. B. *et al.* Lithographically defined porous carbon electrodes. *Small* **5**, 2792-2796 (2009).
