# Supplementary material for: 3D-printed cellular tips for tuning fork atomic force microscopy in shear mode
Source: Nat Commun. 2020 Nov 12;11:5732. doi: 10.1038/s41467-020-19536-9 (PMC7661501; doi:10.1038/s41467-020-19536-9)
Supplement: Supplementary file 3 — Description of Additional Supplementary Files [file 41467_2020_19536_MOESM3_ESM.docx]

**Description of Additional Supplementary Files**

**Supplementary Movie 1:**

Numerically calculated temporal evolutions of indentation depth and stress

distribution in tip-sample impact.
